# The Rho/Rho-associated protein kinase inhibitor fasudil in the protection of endothelial cells against advanced glycation end products through the nuclear factor κB pathway

**DOI:** 10.3892/etm.2013.1125

**Published:** 2013-05-20

**Authors:** YUYAN LU, HAILING LI, WEIXIA JIAN, JIANHUI ZHUANG, KE WANG, WENHUI PENG, YAWEI XU

**Affiliations:** 1 Department of Cardiology, Shanghai Tenth People’s Hospital, Tongji University School of Medicine, Shanghai 200072;; 2 Department of Endocrinology, Xinhua Hospital, Shanghai Jiaotong University School of Medicine, Shanghai 200092, P.R. China

**Keywords:** advanced glycation end products, fasudil, Rho kinase, nuclear factor κB, reactive oxygen species

## Abstract

Accumulating evidence has demonstrated that the Rho/Rho-associated protein kinase (Rho/ROCK) and nuclear factor κB (NF-κB) signaling pathways are involved in the pathogenesis of diabetic vascular injury. In this study, we investigated the beneficial effects of fasudil, a ROCK inhibitor, on vascular endothelial injury induced by advanced glycation end products (AGEs) *in vitro*. Human umbilical vein endothelial cells (HUVECs) were stimulated with AGEs and AGEs plus fasudil in various concentrations for different time periods. Monocyte-endothelial cell adhesion, vascular cell adhesion molecule-1 (VCAM-1) and monocyte chemoattractant protein-1 (MCP-1) expression, protein expression and activation of Rho/ROCK, activation of NF-κB and reactive oxygen species (ROS) production were evaluated. Fasudil suppressed AGE-induced monocyte-endothelial adhesion. Fasudil also reduced the mRNA and protein expression of VCAM-1 and MCP-1 in a concentration- and time-dependent manner. Moreover, increases in the protein levels of Rho/ROCK and ROCK activity mediated by AGEs were inhibited by the addition of fasudil. Additionally, fasudil attenuated AGE-induced NF-κB-dependent transcriptional activity and inhibition of NF-κB (IκB) phosphorylation. ROS production induced by AGEs was also reduced by fasudil in HUVECs. The results suggest that ROCK inhibition may protect the vascular endothelium against AGE-induced monocyte-endothelial adhesion *in vitro* through the reduction of ROS generation and the downregulation of NF-κB signaling. Thus, ROCK inhibition may be a novel therapeutic approach for the treatment of vascular complications in diabetes.

## Introduction

Macro- and micro-vascular complications are the leading causes of high morbidity and mortality in patients with diabetes (1). A causal factor leading to the pathophysiological alterations in the diabetic vasculature is the chronic exposure to advanced glycation end products (AGEs), which result from non-enzymatic glycation and glycoxidation of proteins and lipids when they interact with aldose sugars for an extended period of time (2–4). A study demonstrated that the presence and accumulation of AGEs in endothelial cells affect cell structure and function, thus contributing to the pathophysiology of vascular diseases in diabetes (5). Soluble AGEs activate monocytes, and AGEs in the basement membrane inhibit monocyte migration (5). The migration and adhesion of monocytes to the subendothelial space, mediated by the interaction between monocytes and molecules expressed on the endothelial cell surface (6–8), are partly regulated by adhesion and chemotactic factors, including vascular cell adhesion molecule-1 (VCAM-1) and monocyte chemoattractant protein-1 (MCP-1) (9,10). The expression of VCAM-1 and MCP-1 is mainly mediated by activation of nuclear factor κB (NF-κB) (2,11), which is activated by reactive oxygen species (ROS) (5,12).

Previously, we identified that high glucose levels increase the expression of VCAM-1 and MCP-1 through activation of the RhoA/Rho-associated protein kinase (ROCK) pathway (13). The RhoA/ROCK signaling pathway regulates a wide range of fundamental cellular functions, including cellular apoptosis, metabolism, migration and adhesion (13–17). Regulation of these cellular functions is mainly dependent on the activation of its downstream effector, ROCK (14). There is increasing evidence supporting the hypothesis that ROCK is an important component of signaling pathways involved in the regulation of the inflammatory response (17–20). For example, ROCK has been implicated in the modulation of cell adhesion (13). It has been reported that ROCK inhibition by Y-27632, fasudil or the overexpression of dominant-negative mutants of ROCK, reduces cell adhesion through a loss of focal adhesion complexes and reduced expression of adhesion molecules, including VCAM-1, MCP-1, intracellular adhesion molecule 1 (ICAM-1) and endothelial leukocyte adhesion molecule 1 (13,19,20). In addition, activation of NF-κB is also associated with ROCK (20,21). However, it is unknown whether ROCK inhibition attenuates AGE-induced monocyte-endothelial adhesion through reduction of ROS and downregulation of NF-κB.

Therefore, in the present study, we aimed to investigate the involvement of the Rho/ROCK pathway and NF-κB signaling in the pathogenesis of AGE-induced endothelial injury and the usefulness of fasudil to prevent the disorder *in vitro*.

## Materials and methods

### Preparation of AGEs

AGEs were prepared by the incubation of 50 mg/ml human serum albumin with 1 M glucose in phosphate-buffered saline (PBS; pH 7.4) in the presence of 1.5 mM phenylmethylsulfonyl fluoride (PMSF), 1 mM ethylenediaminetetraacetic acid (EDTA), 100 *μ*g/ml penicillin and 40 *μ*g/ml gentamicin for at least 12 weeks in the dark at 37°C under sterile conditions as previously described (22). Following incubation, unreacted sugar was removed prior to extensive dialysis against PBS. Then, the solution was separated into aliquots and stored at −20°C before use.

### Cell culture

Human umbilical vein endothelial cells (HUVECs) were purchased from Clonetics Cell Discovery Systems (San Diego, CA, USA) and routinely propagated as described previously (13). The human leukemic monocytic cell line THP-1 was obtained from the Institute of Biochemistry and Cell Biology (Shanghai, China). Cells were maintained in RPMI-1640 medium (Gibco-BRL, Grand Island, NY, USA) supplemented with 2 mM L-glutamine and 10% fetal bovine serum.

Following detachment of 90% confluent HUVECs from flasks with 0.025% trypsin, the cells that were derived between the third and tenth passages were plated in 6-well plates. Based on the concentrations of AGEs and fasudil indicated previously (13,23), HUVECs were then stimulated with AGEs or AGEs + fasudil (Tianjin Red Sun Pharmaceutical Co., Ltd., Tianjin, China; 2 ml, 3 mg) for 24 h as follows: i) without any treatment (control); ii) with 200 *μ*g/ml AGEs; iii) with 400 *μ*g/ml AGEs; iv) with AGEs (400 *μ*g/ml) + fasudil (1 nM); and v) with AGEs (400 *μ*g/ml) + fasudil (10 nM). In other experiments, HUVECs were also treated with AGEs (400 *μ*g/ml) or AGEs (400 *μ*g/ml) + fasudil (10 nM) for various times (0, 6, 12, 24 and 48 h). Total RNA and proteins were extracted for further study.

### Analysis of monocyte-endothelial adhesion

Cell adhesion was performed as described previously (19). HUVECs were grown in 6-well plates and pretreated with various concentrations of AGEs or AGEs + fasudil for 12 or 24 h. THP-1 cells passaged regularly were labeled with 10 *μ*g/ml 2′,7′-bis (carboxyethyl)-5(6)-carboxyfluorescein acetoxymethyl ester (BCECF/AM; Sigma, St. Louis, MO, USA) at a final concentration of 10 *μ*M in RPMI-1640 medium for 1 h at 37°C. Then, labeled THP-1 cells were added (1×10^6^ cells/ml) to mono-layers of HUVECs in 6-well plates. When the cells had been incubated for 1 h, a number of bound cells were assayed by fluorescence excitation (488 nm) and emission (535 nm) using fluorescence microscopy (Leica DMI6000, Leica, Wetzlar, Germany) and the number of bound monocytes was calculated by fluorescence intensity. Three fields were captured for each experimental condition and experiments were performed at least three times.

### RNA extraction and quantitative reverse transciption-polymerase chain reaction (RT-PCR)

Total RNA was isolated from HUVECs with TRIzol. RNA (1 *μ*g) was used for first-strand cDNA synthesis with PrimeScript^®^ RT reagent kit (Takara Bio Inc., Otsu, Japan). The mRNA expression was determined by real-time quantitative RT-PCR using the ABI Prism 7900HT (Applied Biosystems, Foster City, CA, USA). Quantitative data of relative gene expression was calculated by the comparative Ct method (^ΔΔ^Ct) with SYBR^®^ Premix Ex Taq™ (Takara Bio Inc.) as described previously (13). Glyceraldehyde 3-phosphate dehydrogenase (GAPDH) was the endogenous control gene.

### Protein extraction and western blot analysis

HUVECs were lysed with ice-cold RIPA and centrifuged at 9,300 × g for 5 min at 4°C. Supernatants were collected and then subjected to sodium dodecyl sulfate-polyacrylamide gel electrophoresis (SDS-PAGE). Western blot analysis was performed using standard methods, with antibodies raised against VCAM-1 (Santa Cruz Biotechnology, Santa Cruz, CA, USA), MCP-1, RhoA, ROCK1, phosphorylated myosin phosphatase target protein 1 (p-MYPT1), phospho-Ser32 inhibitor of NF-κB (IκB) and β-actin (Cell Signaling Technology, Danvers, MA, USA).

### Dual-luciferase reporter assay

The luciferase assay was performed as described previously (24,25). Cells were seeded in 6-well plates for 24 h overnight and transfected with NF-κB promoter-luciferase constructs (Promega, Madison, WI, USA) using Lipofectamine 2000 transfection reagent (Invitrogen Life Technologies, Carlsbad, CA, USA). After serum starvation for 24 h, transfected cells were treated with AGEs or with AGEs + fasudil for 24 h as follows: i) without any treatment (control); ii) with 200 *μ*g/ml AGEs; iii) with 400 *μ*g/ml AGEs; iv) with AGEs (400 *μ*g/ml) + fasudil (1 nM); and v) with AGEs (400 *μ*g/ml) + fasudil (10 nM). Firefly and Renilla luciferase activities in cell extracts were measured using a Dual-Luciferase Reporter Assay system (Promega). The relative activity was calculated by normalizing NF-κB promoter-driven firefly luciferase activity to control Renilla luciferase activity.

### Determination of intracellular ROS

Assay for ROS was performed by measuring superoxide anion (O_2_^−^) release into the supernatant from HUVECs. The detection was based on its ability to cause superoxide dismutase-inhibitable reduction of ferricytochrome *c* (26). Cells were seeded at a density of 1–3×10^6^ cells/ml in 96-well plates overnight, followed by treatment with serum-free media for 24 h. Ferricytochrome *c* (Sigma) was added to a final concentration of 200 *μ*M at room temperature in the presence or absence of 150 U/ml superoxide dismutase (Sigma). HUVECs were pretreated with various concentrations of fasudil for 2 h, followed by exposure to AGEs for 10 min. Reduction of ferricytochrome *c* in the supernatant was measured by reading the absorbance at 550 nm in a spectrophotometer. The amount of O_2_^−^ release was calculated by dividing the difference in absorbance of the samples, with and without superoxide dismutase. The results are expressed in nmol/ml.

### Statistical analysis

Data are expressed as mean ± standard deviation (SD). Comparison between groups was performed using one-way analysis of variance (ANOVA), followed by Bonferroni multiple comparison test. P<0.05 was considered to indicate a statistically significant difference. All statistical analyses were performed with SPSS for Windows 13.0 (SPSS, Inc., Chicago, IL, USA).

## Results

### Fasudil inhibits the AGE-induced cell adhesion in vitro

**Figure 1.**
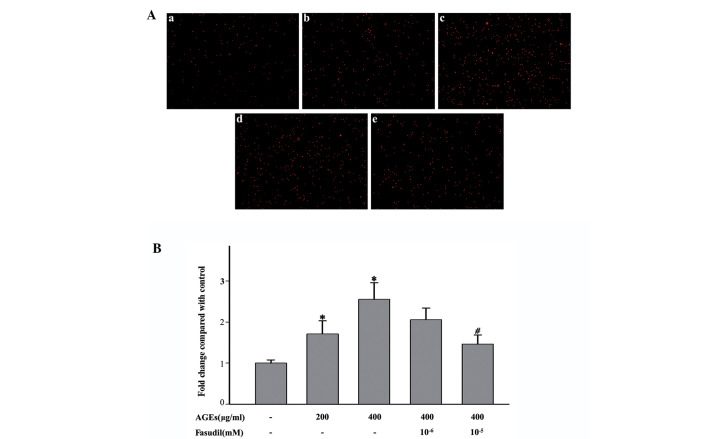
Effects of fasudil on advanced glycation end-product (AGE)-induced monocyte-endothelial cell adhesion. (A) Human umbilical vein endothelial cells (HUVECs) and THP-1 cells labeled with BCECF/AM were grown in 6-well plates and stimulated with AGEs or AGEs + fasudil for 12 h. (a) Control, (b) 200 *μ*g/ml AGEs, (c) 400 *μ*g/ml AGEs, (d) AGEs(400 *μ*g/ml) + fasudil (1 nM), (e) AGEs (400 *μ*g/ml) + fasudil (10 nM). (B) Results expressed as fluorescence fold induction compared with control. Values are presented as mean ± standard deviation (SD) of at least three experiments. ^*^P<0.05 vs. control; ^#^P<0.05 vs. AGEs (400 *μ*g/ml) alone.

The effect of fasudil on cell adhesion was evaluated with BCECF/AM-labeled monocytes. Incubation with AGEs for 12 h significantly increased the adhesion of THP-1 cells to HUVECs compared with the control group (Fig. 1; incubation with 200 *μ*g/ml and 400 *μ*g/ml AGEs caused 1.7- and 2.5-fold changes, respectively; both P<0.05). Additionally, we assessed the effect of fasudil on AGE-induced cell adhesion. HUVECs treated with fasudil resulted in a suppression of cell adhesion (treatment with 1 and 10 nM fasudil reduced cell adhesion by ∼16 and 43%; P<0.10 and P<0.05, respectively; Fig. 1). Similar results were observed with 24 h incubation (data not shown).

### Effects of fasudil on the expression of VCAM-1 and MCP-1

**Figure 2.**
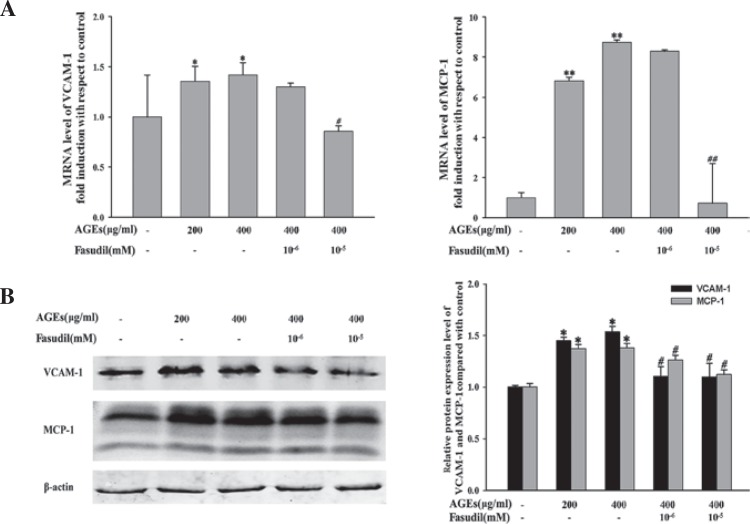
Dose-dependent effects of advanced glycation end-products (AGEs) and fasudil on the expression of vascular cell adhesion molecule-1 (VCAM-1) and monocyte chemoattractant protein-1 (MCP-1) in human umbilical vein endothelial cells (HUVECs). For all experiments, HUVECs were serum-starved and exposed to AGEs or AGEs + fasudil at the indicated dose for 24 h. VCAM-1 and MCP-1 mRNA levels were assayed with quantitative reverse transcription polymerase chain reaction (RT-PCR). Protein expression levels of VCAM-1 and MCP-1 were determined by western blot analysis. Each band density was normalized to its own control. (A) Effects of AGEs and fasudil on the mRNA expression of VCAM-1 and MCP-1 contrasted by dose. (B Effects of AGEs and fasudil on the protein expression of VCAM-1 and MCP-1 for indicated doses. Values are presented as mean ± standard deviation (SD). ^*^P<0.05, ^**^P<0.01 vs. control; ^#^P<0.05, ^##^P<0.01 vs. 400 *μ*g/ml AGEs alone.

**Figure 3.**
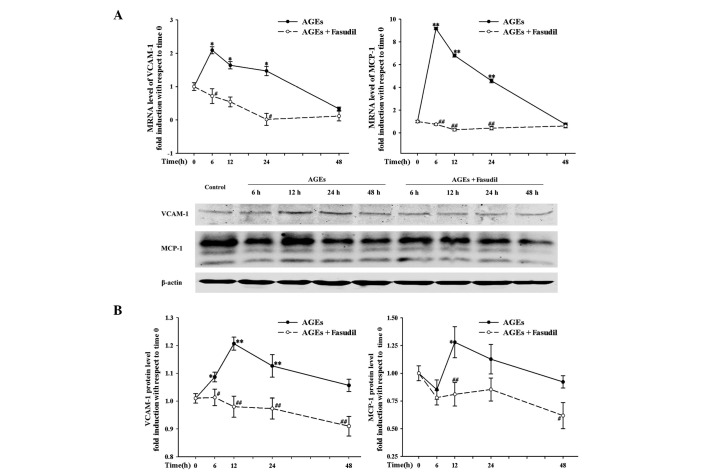
Time-dependent effects of advanced glycation end-products (AGEs) and fasudil on vascular cell adhesion molecule-1 (VCAM-1) and monocyte chemoattractant protein-1 (MCP-1) expression in human umbilical vein endothelial cells (HUVECs). HUVECs were treated with AGEs (400 *μ*g/ml) or AGEs (400 *μ*g/ml) + fasudil (10^−5^ mM) for various times (0, 6, 12, 24 and 48 h). The mRNA and protein expression levels were measured. (A) Time course of mRNA levels of VCAM-1 and MCP-1. (B) Time course of protein expression of VCAM-1 and MCP-1. ^*^P<0.05, ^**^P<0.01 vs. time 0; ^#^P<0.05, ^##^P<0.01 vs. AGEs (400 *μ*g/ml).

To elucidate the underlying mechanism of monocyte-endothelial adhesion, we determined the effects of AGEs and fasudil on the expression of VCAM-1 and MCP-1. As shown in Fig. 2, HUVECs incubated with AGEs (200 or 400 *μ*g/ml) for 24 h demonstrated increased mRNA (Fig. 2A) and protein (Fig. 2B) expression levels of VCAM-1 and MCP-1, particularly MCP-1. Fasudil (10 or 1 nM) attenuates the expression of adhesion and chemotactic factors. There were no significance differences in the HUVECs incubated with fasudil at a low dose (1 nM), suggesting that fasudil at a high dose had greater effects on HUVECs compared with at a low dose. Moreover, Fig. 3 shows the mRNA (Fig. 3A) and protein (Fig. 3B) expression of HUVECs treated with AGEs (400 *μ*g/ml) and AGEs (400 *μ*g/ml) + fasudil (10 nM) for various times. At corresponding time-points, the mRNA and protein expression levels of these factors were significantly lower with fasudil treatment than without fasudil treatment. These results suggest that fasudil inhibited AGE-induced cell adhesion by reducing the expression of these adhesion and chemotactic factors.

### Fasudil suppresses ROCK activity and the protein levels of Rho/ROCK

**Figure 4.**
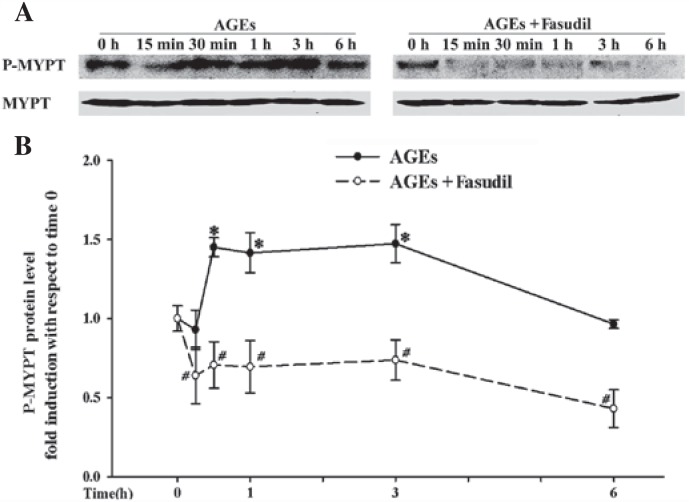
Effects of advanced glycation end-products (AGEs) and fasudil on Rho-associated protein kinase (ROCK) activity in human umbilical vein endothelial cells (HUVECs). (A) HUVECs were stimulated with AGEs or AGEs + fasudil for the indicated times (0, 15 min, 30 min, 1 h, 3 h and 6 h). Expression of phosphorylated myosin phosphatase target (p-MYPT)-1 and MYPT-1 proteins was measured by western blot analysis. Results are expressed as fold induction with respect to time 0. (B) Data are presented as mean ± standard deviation (SD). ^*^P<0.05 vs. time 0; ^#^P<0.05 vs. AGEs (400 *μ*g/ml).

To ascertain whether AGEs increased cell adhesion via the Rho/ROCK pathway, we assessed the levels of p-MYPT1, as described in previous studies (13,27). In the present study, HUVEC exposure to AGEs resulted in an increased p-MYPT1/MYPT1 ratio from 15 min to 3 h compared with the control groups, indicating that AGEs activate the Rho/ROCK pathway (Fig. 4). As expected, fasudil demonstrated inhibitory effects on AGE-induced ROCK activation over the experimental period.

**Figure 5.**
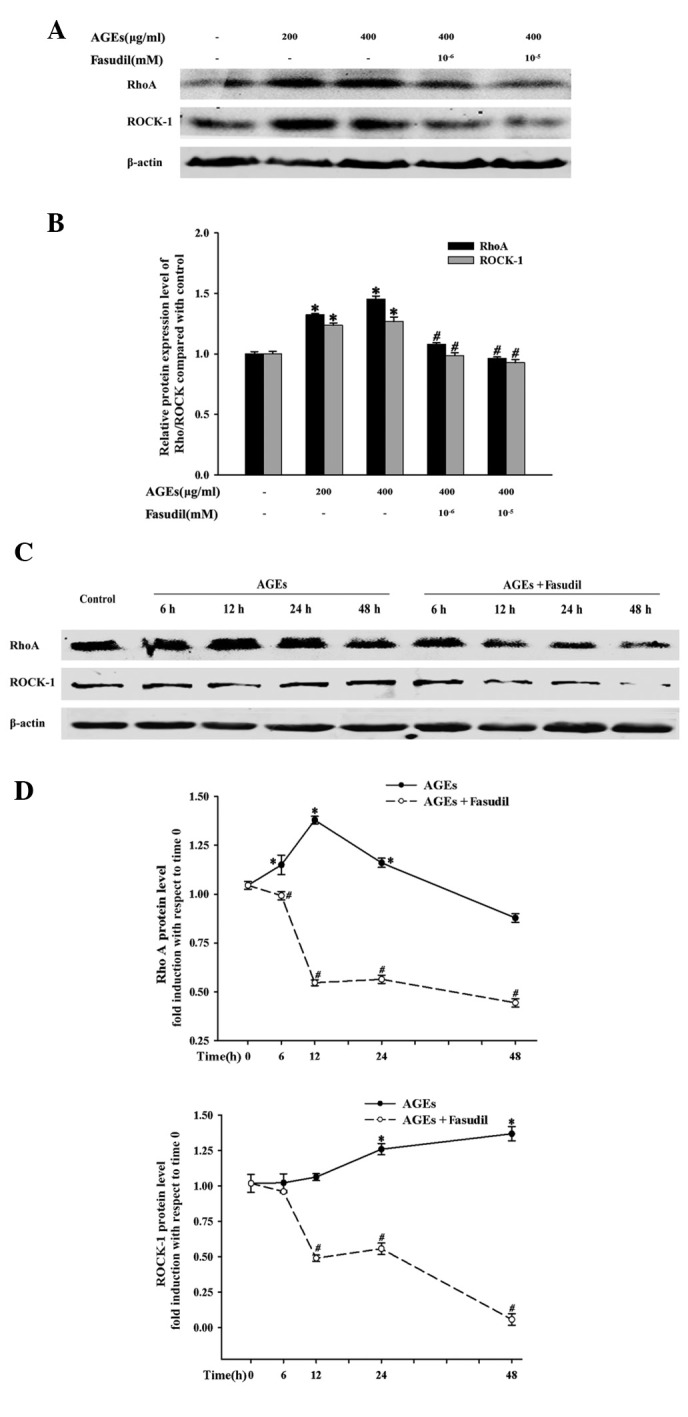
Effects of advanced glycation end-products (AGEs) and fasudil on Rho/Rho-associated protein kinase (ROCK) protein expression. RhoA and ROCK-1 proteins were measured by western blotting. Each band density was normalized to its own control. (A and C) Dose- and time-dependent effects of AGEs and fasudil on human umbilical vein endothelial cells (HUVECs). (B) Summarized data (mean ± SD) from the dose-dependent experiments. *P<0.05 vs. control; ^#^P<0.05 vs. AGEs (400 *μ*g/ml) alone. (D) Summarized data (means ± SD) from the time-dependent experiments. ^*^P<0.05 vs. time 0; ^#^P<0.05 vs. AGEs (400 *μ*g/ml). All data shown are representative of 3–4 independent experiments. SD, standard deviation.

In addition, exposure to AGEs increased the protein expression levels of RhoA and ROCK1 in a concentration- and time-dependent manner (Fig. 5). However, the effects were attenuated by the addition of fasudil. In the presence of 10 nM fasudil, the AGE-induced protein expression levels were significantly reduced from 6 to 48 h.

### Fasudil attenuates AGE-induced NF-κB activation and ROS

**Figure 6.**
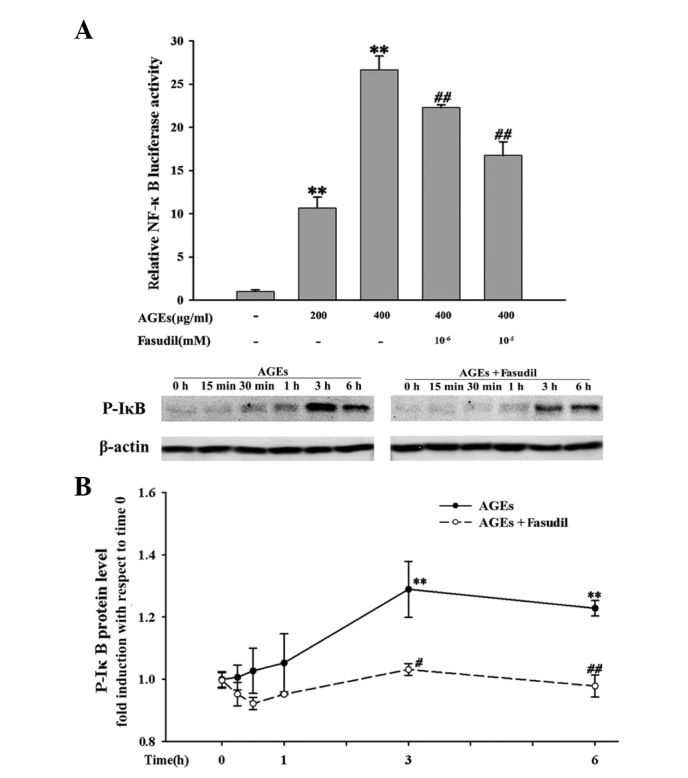
Effects of advanced glycation end-products (AGEs) and fasudil on nuclear factor (NF)-κB activation. (A) Effects of fasudil on NF-κB-dependent transcriptional activity with a NF-κB-luciferase reporter plasmid transiently transfected in human umbilical vein endothelial cells (HUVECs) at 24 h. Results are expressed as fold induction with respect to the control. ^**^P<0.01 vs. control; ^##^P<0.01 vs. AGEs (400 *μ*g/ml) alone. (B) The time-dependent effects of fasudil on inhibitor of NF-κB (IκB) phosphorylation, measured by western blotting, and summarized data (mean ± SD). Results are expressed as fold induction with respect to time 0. ^**^P<0.01 vs. time 0; ^#^P<0.05, ^##^P<0.01 vs. AGEs (400 *μ*g/ml). SD, standard deviation.

In order to investigate whether ROCK is involved in AGE-induced NF-κB activation, we evaluated the effects of fasudil on NF-κB-dependent transcriptional activity with a NF-κB-luciferase reporter plasmid transiently transfected in HUVECs. As shown in Fig. 6A, AGEs significantly increase NF-κB-dependent transcriptional activity in a concentration-dependent manner at 24 h (P<0.01) and fasudil significantly inhibited the increase. Since IκB phosphorylation plays an important role in the activation of NF-κB, we next evaluated the effects of fasudil on IκB phosphorylation to further investigate the molecular target of fasudil in NF-κB signaling. Fasudil attenuated AGE-induced IκB phosphorylation from 3 to 6 h (Fig. 6B). These results demonstrated that ROCK is involved in the pathway that activates NF-κB signaling.

**Figure 7.**
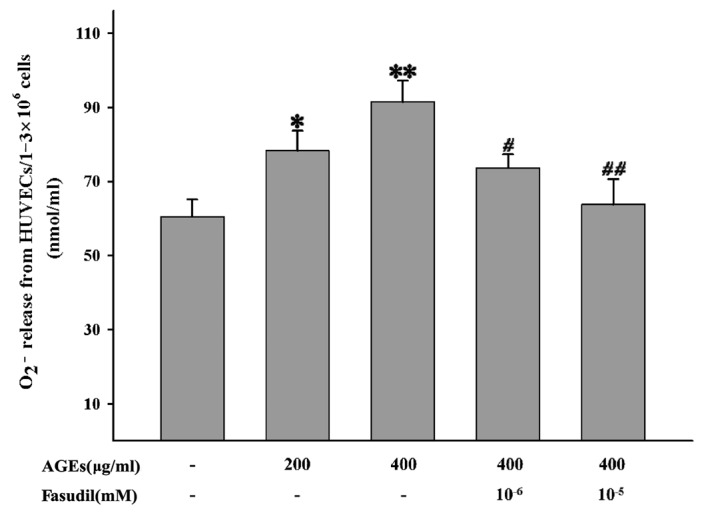
Effects of advanced glycation end-products (AGEs) and fasudil on reactive oxygen species (ROS). Superoxide anion (O_2_^−^) release into the supernatant from human umbilical vein endothelial cells (HUVECs) was measured by reduction of ferricytochrome *c*. Results are expressed as nmol/ml. Data are presented as means ± standard deviation (SD). ^*^P<0.05, **P<0.01 vs. control; ^#^P<0.05, ^##^P<0.01 vs. AGEs (400*μ*g/ml) alone.

Additionally, to assess the effects of fasudil on ROS production, we further studied O_2_^−^ release into the super-natant from HUVECs. As shown in Fig. 7, AGEs promote O_2_^−^ release; a significant increase in the reduction of ferricytochrome *c* was observed in comparison with the control groups, particularly at a high concentration. However, the effects of AGEs on O_2_^−^ release were successfully inhibited by the addition of fasudil. These results suggest that fasudil significantly inhibited ROS production and that a high dose of this agent had more potent inhibitory effects. These data together demonstrated that fasudil inhibited ROS generation from HUVECs in response to AGEs and then inhibited the activation of NF-κB.

## Discussion

Major findings from this study demonstrated that i) fasudil protected the vascular endothelial cells against AGEs-induced adhesion of monocytes to the endothelium, and ii) the effects of fasudil with regard to the inhibition of cell adhesion were partly due to the reduction of ROS production and inhibition of the Rho/ROCK and NF-κB signaling pathways. Our study suggests that fasudil plays a role in the protection of the vascular endothelium through inhibition of the Rho/ROCK pathway, reduction of ROS generation and downregulation of NF-κB signaling. Such a phenomenon may provide insights into molecular mechanisms of vascular protection in diabetes.

As indicated previously, a notable feature of the complicated inflammation process in the vasculature of diabetics is monocyte-endothelial adhesion (6), which is induced partly by AGEs through adhesion molecules, including VCAM-1 and ICAM-1 (5). Thus, it is necessary to identify effective therapies that inhibit AGE-induced cell adhesion in diabetes; however, related treatment for this aspect is limited. Our previous study suggested that ROCK inhibition may have therapeutic effects in preventing high glucose-associated vascular inflammation and atherogenesis (13). In line with our previous study (13), fasudil markedly reduced AGE-induced cell adhesion by reducing the mRNA and protein expression levels of VCAM-1 and MCP-1 in HUVECs, and fasudil at a high dose (10 nM) provided superior efficacy. The exposure of HUVECs to AGEs increased the protein expression of Rho/ROCK and activated MYPT phosphorylation. Simultaneously, the effects were significantly suppressed by fasudil. These results suggest that the Rho/ROCK pathway was involved in the progression of AGE-induced cell adhesion.

Since VCAM-1 and MCP-1 expression in response to AGEs has been reported to be regulated by NF-κB signaling, we investigated the association between ROCK inhibition and NF-κB signaling. In the present study, we identified that treatment of HUVECs with fasudil successfully inhibited AGE-induced NF-κB activity and simultaneously decreased IκB phosphorylation. There are also several lines of evidence indicating that ROCK is involved in the pathway that activates NF-κB; however, the role of the Rho/ROCK pathway in NF-κB signaling remains inconsistent and may vary depending on activation stimulus. Bolick *et al* reported that NF-κB is activated in the endothelial cells of 12/15-lipoxygenase transgenic mice and that ROCK inhibition blocked NF-κB activation and monocyte adhesion (28). Moreover, thrombin and interleukin 1β (IL-1β) were shown to increase ROCK activity, the transcriptional activation of NF-κB and then adhesion molecule expression (20,29,30). However, in parallel studies, researchers failed to observe the inhibitory effects on NF-κB activation in response to tumor necrosis factor α (TNF-α) and lipopolysaccharide (LPS) (29,30). By contrast, our findings revealed that ROCK was involved in AGE-induced NF-κB activation and the expression of adhesion molecules.

Furthermore, increasing evidence indicates that ROS also plays an important role in the pathophysiology of diabetic vascular endothelial injury through NF-κB activation (5,31) and ROCK enhances the production of ROS via activation of nicotinamide adenine dinucleotide phosphate (NADPH) oxidase (32,33). However, ROCK involvement in NADPH oxidase activation following the stimulation of cultured HUVECs by AGEs remains to be identified. Our results confirmed that ROCK activation was involved in superoxide formation. AGE administration to HUVECs *in vitro* caused increased ROS production and NF-κB activation. Treatment with fasudil significantly attenuated ROS formation. Therefore, we may infer that ROCK inhibition plays a role in suppressing NF-κB activity via ROS production.

In summary, our findings indicate that fasudil attenuates AGE-induced monocyte-endothelial cell adhesion and the expression of VCAM-1 and MCP-1 through the Rho/ROCK pathway. In addition, the inhibitory effects of fasudil on AGE-induced inflammatory responses were partly due to the reduction of ROS production and inhibition of NF-κB.
